# Electron pair escape from fullerene cage via collective modes

**DOI:** 10.1038/srep24396

**Published:** 2016-04-18

**Authors:** Michael Schüler, Yaroslav Pavlyukh, Paolo Bolognesi, Lorenzo Avaldi, Jamal Berakdar

**Affiliations:** 1 Institut für Physik, Martin-Luther-Universität Halle-Wittenberg, 06099 Halle, Germany; 2 CNR-ISM, Area della Ricerca di Roma 1, CP10, 00016 Monterotondo Scalo, Italy

## Abstract

Experiment and theory evidence a new pathway for correlated two-electron release from many-body compounds following collective excitation by a single photon. Using nonequilibrium Green’s function approach we trace plasmon oscillations as the key ingredient of the effective electron-electron interaction that governs the correlated pair emission in a dynamic many-body environment. Results from a full *ab initio* implementation for C_60_ fullerene are in line with experimental observations. The findings endorse the correlated two-electron photoemission as a powerful tool to access electronic correlation in complex systems.

A sample absorbing a single ultraviolet photon may emit a single electron having energy and momentum distributions that reflect the spectral properties of the material^1^. It is also possible, though usually less probable, that two electrons escape. How can one photon “kick out” two electrons? For few-electron atoms, it is established^2^ that the Coulomb repulsion plays a key role. A possible scenario is that the photon is absorbed by one bound electron that approaches the other electron while undergoing multiple scattering from the residual ion or the other electron. Mediated by electron-electron Coulomb interaction, the two electrons exchange momentum while leaving the sample and interacting mutually and with the residual ion, in principle to infinite distances. This physical picture, often referred to as *knock-out* mechanism, dominates for photon energies close to double ionization threshold, whereas for larger photon energies different processes (e.g. *shake-off* ) become important^3^. When detecting the two electrons in coincidence (called double photoemission (DPE) spectroscopy^4^,^5^,^6^), depending on the selected energies and angles, one may zoom into some of these processes, albeit with restrictions imposed by symmetry^2^,^7^.

The situation changes with a growing number of electrons in the system. The effective electron-electron interaction is not even known *a priori* as it is determined by the dynamic behavior of its active surrounding, meaning that the *e–e* interaction builds up during the photoexcitation process. Thereby, dimensionality is a key factor^8^. In fact, for electronic systems strongly confined to one dimension (e.g., a one channel quantum wire) *e-e* interaction gives rise to a new form of excitations (Luttinger liquid^9^) with features distinct from those akin to Fermi liquids, i.e. most three-dimensional systems. As DPE experiments are available for weakly and moderately correlated surfaces and bulk materials (e.g. Cu, NiO or CoO^10^,^11^,^12^), it is valuable to consider DPE for nano-sized systems that bridges the extended and atomic cases.

A possible scenario of DPE is that the photon excites one electron which senses its environment for accessible scattering channels (elastic, phononic, magnonic, etc). DPE at a fixed incident photon energy via the selection of the energy sharing and relative angles between the two escaping electrons zooms into those channels, where electron-electron (*e–e*) interaction is operational. The focus here is on *e–e* interaction mediated by charge density fluctuations in confined geometry. On the other hand, electronic correlations are at the heart of diverse fundamental phenomena such as superconductivity and plasmon formation which underlines the relevance of the information encoded in the DPE spectra. Theoretically, the treatment of two-particle correlations is a central problem in many-body physics^13^,^14^,^15^,^16^.

For the electron gas in particular, focus was put on two aspects affecting the two-particle interaction. 1) Long wave-length density fluctuations which are characterized by the presence of classical excitations (plasmons) and are well captured, for instance by the time-dependent density functional theory (TDDFT) or the random phase approximation^17^,^18^. 2) Short wave-length effects (exhibited in the on-top pair distribution function^19^,^20^,^21^) which are captured by the ladder diagrams^22^,^23^. Exploiting the tunability of synchrotron radiation, DPE (cf. Fig. 1(a)) can be tuned to an energy region where the dynamic and non-local field of collective excitations (plasmons) is the main driving for secondary electron emission whilst short-range effects govern the formation of two-particle scattering states.

A standard single photoemission (SPE) theory usually relies on the hole spectral density, which accommodates so-called *intrinsic* energy losses, and the optical matrix elements. Plasmon-mediated processes are typical for *extrinsic* losses. These refer to all scattering events which the photoelectron undergoes before detection^24^. Formulating a theory for SPE valid for all types of electronic systems, proved to be an involved task. The perturbation theory for the transition dipole, as employed for atoms or molecules^25^ is in principle able to incorporate both electron-electron scattering processes and also collective effects^26^. One may also attempt at a direct diagrammatic expansion of the observable photocurrent, as was put forward in ref. 27. A formal theory of DPE entails the use of many-body perturbation theory (MBPT) for two-particle propagators^15^ and is thus even more involved. Based on the direct diagrammatic approach for the observable coincidence yield^28^ we present here the first fully *ab initio* implementation for DPE accompanied by charge density fluctuations and compare with the first experiments of this kind on C_60_. Our approach is applicable to complex atoms such as Xe possessing strong collective resonances^29^, as well.

The emerging physical picture is illustrated in Fig. 2(a): (i) Photoabsorption promotes a valence electron to a high-energy state. (ii) This electron scatters inelastically from charge-density fluctuations (plasmon creation) that (iii) decay emitting a second valence electron (whose energy and angular correlations with the first one is measured in a coincidence set up, revealing so how charge-density fluctuations mediate *e–e* interaction). This three-step mechanism (3SM) emerges from a diagrammatic nonequilibrium Green’s function (NEGF) approach as detailed in the supplementary information. It is already clear at this stage that DPE is qualitatively different from SPE in that, a) it delivers information on *e–e* interaction mediated by charge-density fluctuations, and b) as these plasmonic excitations are triggered by an electron a multitude of modes, e.g. volume plasmons, are involved.

## Results

**Figure 1:**
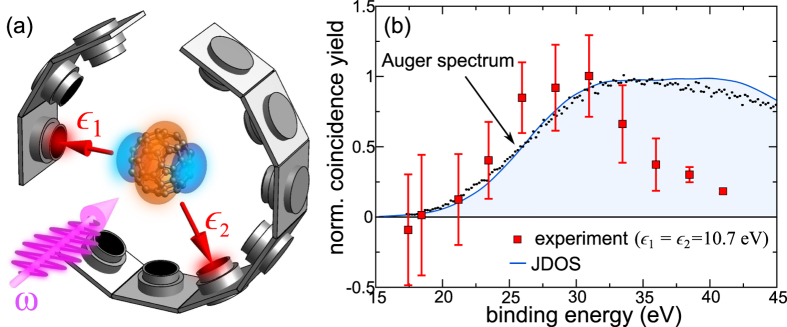
(**a**) DPE Setup: upon absorbing one photon with energy 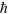
ω, two correlated electrons are emitted non-sequentially from the C_60_ molecule and detected in coincidence. Charge-density fluctuations play the key role for the correlation hereby. (**b**) For equal energies of the emitted electrons 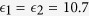 eV, the normalized coincidence yield versus C_60_ binding energy (red squares with error bars) is compared to the Auger spectrum with 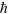
ω = 340 eV (black dots). The latter is compared to our calculations of the joint density of states (JDOS) (shaded blue line).

**Figure 2:**
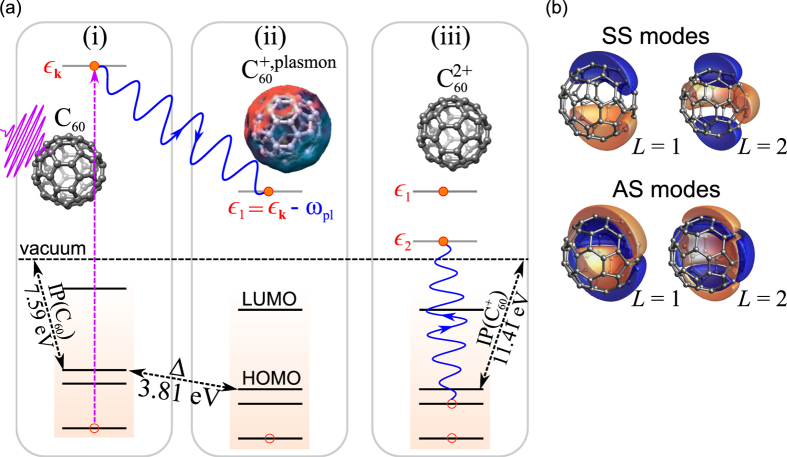
(**a**) Energy level scheme for DPE mediated by charge-density fluctuations in three steps: (i) A valence electron is photo-promoted to an intermediate state with energy 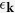. (ii) This electron scatters inelastically from excited C_60_ to an energy state 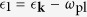 while creating multipolar plasmonic modes with energy *ω*_pl_ that (iii) decay on the attosecond time scale^52^, leading to the coherent emission of a second electron (energy 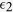) if *ω*_pl_ is larger than the ionization potential (IP) of 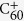. (**b**) A cut through fullerene center of the calculated fluctuation densities *ρ*_*νLM*_(**r**) of the symmetric (SS) and anti-symmetric (AS) surface plasmon modes. *L*(*M*) characterizes the multipolarity (and its azimuthal behavior) and *ν* is a radial quantum number (here *M* = 0). Colored regions represent *ρ*_*νLM*_(**r**) > 5 × 10^−4^ a.u. (light orange) and *ρ*_*νLM*_(**r**) < −5 × 10^−4^ a.u. (dark blue).

In Fig. 1(b) the electron pair coincidence yield versus the binding energy 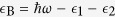 of the doubly charged ion is reported and compared with the Auger spectrum. The binding energy of the latter is determined by the energy of the secondary electron and the carbon 1 s core level (see methods). The Auger process, which one might expect to be comparable to DPE when plotting as a function of the binding energy, can be interpreted in terms of the joint density of states (JDOS) as determined by the convolution of the density of occupied states of the neutral system, 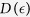, and that of the ionized molecule, 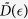, 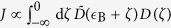. Our *ab initio* calculations in Fig. 1(b) confirm this picture (note, these same 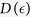 and 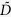 are also part of DPE and are calculated with the same code). For plasmon-mediated DPE the situation is different. As inferred from Fig. 2(a), (ii), the spectral width of the plasmon modes is a determining factor for the width of the DPE spectrum. Which mode is active (and what is its multipolar nature) is set by the momentum balance that in turn points to the momentum region of the involved plasmons. The full *ab initio* calculations of multipolar plasmons in C_60_ in ref. 30 enter as a part (i.e. steps (ii)*–*(iii) Fig. 2(a)) of our DPE calculations.

The electron pair coincidence yield is calculated following the derivation in the supplementary information. From Fig. 2 one infers that the non-local, frequency dependent screened electron-electron interaction *W* = *v* + *vχv* is a central quantity for DPE (*v* is the bare Coulomb interaction). As expected from the scheme in Fig. 2 the density-density response function *χ*(**r**, **r′**; *ξ*) is also the key factor for the electron energy loss experiments^31^,^32^,^33^ and also for the screening of the optical field^34^,^35^ by charge-density fluctuations in SPE (in our experiment this effect is negligible because the optical frequency is higher than the relevant plasmon resonances). We write the effective *e-e* interaction in the form


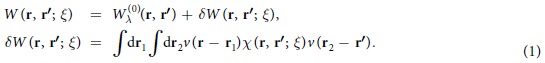


Here, *ξ* denotes the frequency dependence, while *λ* represents a screening parameter discussed below. The collective modes are well characterized by the multipolarity *L* and a radial quantum number *ν*^30^,^36^. We account for symmetric surface (SS), the anti-symmetric surface (AS) and volume (V) modes (*L* = 0). The Lehmann representation of the response function is expressible as


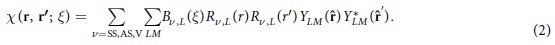



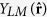
 are the spherical harmonics. 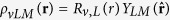 are known as fluctuation densities, which can be interpreted as the spatial distribution of the density oscillation associated to a particular plasmon (Fig. 2(b)). The corresponding frequency spectrum is represented by *B*_*ν*,*L*_(*ξ*). For the radial profiles *R*_*ν*,*L*_(*r*) and plasmon spectra *B*_*ν*,*L*_(*ξ*) we utilize our recent approach from ref. 30 that yielded EELS spectra in very good agreement to experiments^33^. The static part in eq. (1) is written as 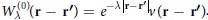. Previous calculations^37^ provided an insight into the value of *λ*. The two-electron coincidence yield, averaged over the initial orientations of C_60_, reads


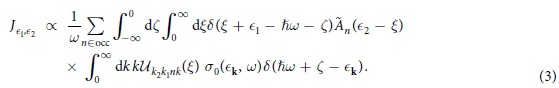


Here, 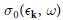 is the partial single-ionization cross section for a photoelectron with energy 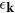. The momenta of the two photoelectrons are denoted by *k*_1_ and *k*_2_. The sum over *n* runs over all occupied states of the singly ionized molecule and 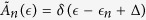 is the corresponding spectral function. 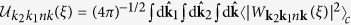 is proportional to the angle-integrated and orientation-averaged (indicated by 〈…〉_*c*_) electron-impact ionization cross section^25^,^38^ as calculated from the two-body matrix elements 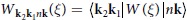. Inspecting eq. (3) one identifies the steps (i)*–*(iii) sketched in Fig. 2(a). Note, due to rearrangement of the ionic core, the energy levels of the neutral molecule (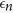) are lowered by Δ when removing one electron, such that the IP increases [see Fig. 2(a)]. *Overall* energy conservation follows from the restrictions (i) 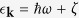, (ii) 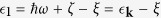, and (iii) 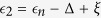.

**Figure 3:**
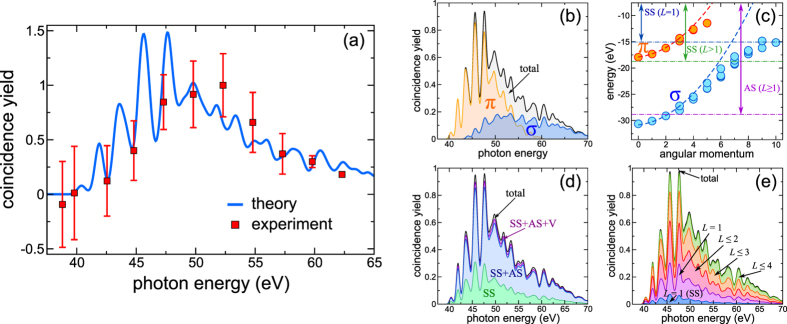
(**a**) Comparison of theoretical prediction of the coincidence spectrum to experimental data. Curves have been normalized to each others at one point. Gaussian broadening of the electronic states is set to *η* = 0.25 eV. (**b**) Normalized coincidence yield resolved in emission of the second electron from *σ* and *π* states, respectively. (**c**) Single-particle energies of the 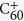 molecule as function of the dominant angular momentum. Dot-dashed lines: accessible energy range of the plasmon modes (FWHM of the plasmon spectra *B*_*ν*,*L*_(*ξ*), shifted by the photoelectron energy 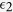). Thick dashed lines: ideal dispersion for non-interacting particles on a sphere with radius *R*_0_ = 3.57 Å. Coincidence yield resolved with respect to plasmon modes (**d**), and multipolarity (**e**).

The computed coincidence photocurrent for the experimental setup of 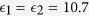 eV is presented in Fig. 3(a) along with the data from the experiment. The equal energy-sharing case has been chosen by the experience on atoms, where this represents the case where the effects of the correlation and symmetry play a dominant role. Further tests (see supplementary information) show that, in contrast to the Auger process [Fig. 1(b)], all ingredients of Eq. (3) (and hence all steps in Fig. 2) are essential: matrix elements effects encoded in 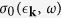, plasmon dispersions *B*_*ν*,*L*_(*ξ*), radial profiles of the fluctuations densities *R*_*ν*,*L*_(*r*), and density of states 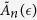. Hence, DPE in the present case adds new aspects to DPE from, e.g., atomic targets, and is a useful sensor for the *e–e* interaction mediated by charge-density fluctuations.

## Discussion

The mechanism behind the narrowing of the DPE as compared to the Auger spectrum [Fig. 1(b)] can be unraveled by analyzing the electronic structure and the individual plasmon modes as they contribute to DPE [Fig. 3(b–e)]. Resolving the DPE yield with respect to either *σ* or *π* orbitals^39^ [Fig. 3(b)] one realizes that the emission from the *σ* band [Fig. 3(c)], which is mainly responsible for the DPE signal at photon energies 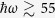 eV, is suppressed by the energy selectivity of the plasmon excitation. In particular, the plasmon giving rise to the emission of the second electron at stage (iii) needs to provide sufficient energy to promote a certain initial state of the 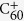 molecule [Fig. 3(c)] to the continuum. Hence, the limited spectral width of the SS plasmon modes suppresses the emission from deeper *σ* states [Fig. 3(b)]. A test calculation replacing the plasmon spectra *B*_*ν*,*L*_(*ξ*) by a constant produced a significantly broader DPE spectrum. This confirms the picture outlined above.

Our theory permits also to selectively include different plasmonic modes in the calculation. It is known that plasmon excitation upon photoabsorption obeys optical dipole selection rules and allows for exciting the SS plasmon with *L* = 1 only (the dipolar resonance mostly addressed in the literature and manifested e.g. in plasmon-assisted SPE^40^). On the contrary, the electron scattering (as in EELS) transfers a finite momentum meaning that SS and AS plasmons with any multipolarity can be excited^30^,^33^,^36^. In agreement with this Fig. 3(d) underpins the significant contribution of the AS plasmons and thus substantiates the physical picture of the 3SM, according to which the step ii) can be regarded as an inelastic electron scattering event that is inherent to the plasmon-assisted DPE process. Similarly, SS dipolar plasmon transitions play only a minor role, while the multipolar plasmons are responsible to a large extent for the coincidence yield [Fig. 3(e)]. All these facets endorse that DPE mediated by charge-density fluctuations as the predominant channel for *e-e* correlations represents a new facet to the information what is extractable from SPE and Auger spectra.

To summarize, an *ab initio* scheme for this process has been implemented with results in line with the first DPE experiment resolved with respect to the electron pair energies. We identified the dominant pathway as the following: a valence electron absorbs the photon and rescatters inelastically from *multipolar* collective modes that mediate the coherent emission of a second electron. The dwell time for this quasi-resonant scattering may be accessed by attosecond time-delay experiments^41^. For plasmon-assisted DPE the average electronic density plays a decisive role. For metals the plasmonic energies (which can be estimated using a classical expression 
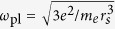
 with *r*_*s*_ being the Wigner-Seitz radius) are too low for plasmons to lead to a direct electron emission, although these modes may likely contribute to the loss channel for DPE. In contrast, for confined systems such as Carbon-based fullerenes the density is much higher (*r*_*s*_ ≈ 1.0 a_B_) resulting in plasmonic peaks in the XUV range. Thus, energy- and angle resolved DPE experiments open the opportunity to explore different regimes of electronic correlation, including Coulombic scattering, local field effects and dynamical screening.

## Methods

### Experiment

The experiments have been performed using the multi-coincidence end station^42^ of the Gas Phase Photoemission beam line^43^ at Elettra, where fully linearly polarized radiation in the photon energy range 13–1000 eV is available. The vacuum chamber hosts two independent turntables, holding respectively three and seven electrostatic hemispherical analyzers at 30° with respect to each other (Fig. 1(a)). The three spectrometers of the smaller turntable, are mounted at 0°, 30°, and 60° with respect to the polarization vector of the light in the plane perpendicular to the propagation direction of the radiation. The larger turntable rotates in the same plane and its seven analyzers can be used to measure the angular distribution of the correlated electrons. The ten analyzers have been set to detect electrons of kinetic energy 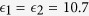 eV. The energy resolution and the angular acceptance were 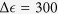 meV and Δ*θ*_1,2_ = ±3°, respectively. The photon energy resolution was about 150 meV. At variance with previous works^44^,^45^,^46^ where the di-cation yield was measured versus photon energy, here the energy spectrum of the C_60_ di-cation states is reconstructed by detection of photoelectron-photoelectron pairs in coincidence as the photon energy is scanned. In order to improve the statistical accuracy of the experimental results, the coincidence signals were added up, after a careful energy calibration of the non-coincidence spectra independently collected by the ten analyzers. The C_60_ source is collinear with the photon beam^47^, which passes through the hollow core of the source before interacting with the molecular beam and ending up on the photodiode. Six apertures drilled into the closure piece of the crucible and pointing to the interaction region increase the molecule density therein.

In the Auger measurements the photon energy was fixed at 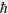
ω = 340 eV and Auger electrons with kinetic energy 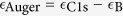, where 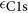 is the binding energy of the carbon 1 s core state and 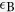 stands for the binding energy in Fig. 1(b) ranging from 15 to 45 eV, were measured.

### Theory

**Figure 4:**
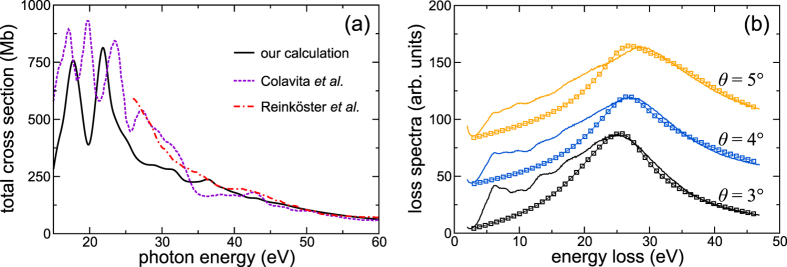
(**a**) Comparison of our calculation of the total SPE cross section with the calculations from Colavita *et al.*^50^ and the experiment from Reinköster *et al.*^51^ on the absolute scale. (**b**) EELS spectra computed with our model response function eq. (2) (symbols) compared to experimental data^33^ (solid lines) for different scattering angles *θ*. The prefactor between theory and experiment was fixed for *θ* = 3° and kept constant for *θ* = 4° and *θ* = 5°.

Equation (3) is derived from the diagrammatic approach to photoemission^28^ based on the nonequilibrium Green’s function formalism. The full derivation is presented in the supplementary information. For an *ab initio* implementation of eq. (3) we rely on density functional theory (DFT) to compute the Kohn-Sham (KS) bound orbitals and their energies 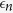. We used the local density approximation (LDA) with self-interaction corrections. They improve the asymptotic behavior of the KS potential that is utilized to compute scattering states. The IPs and the core rearrangement shift Δ enter as experimentally determined^44^,^48^. The SPE cross section 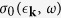 is computed by the driven-scattering approach^49^, yielding excellent agreement with literature data^50^,^51^ in the relevant energy range [Fig. 4(a)] of 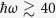 eV. Note that incorporating many-body effects is not required here (as they mainly influence the cross section around the plasmon resonances). The multipolar plasmon modes entering eq. (2) needed for computing the effective interaction (1) is parameterized according to previous calculations^30^ and tested against EELS measurements in Fig. 4(b). Describing the Auger spectrum in Fig. 1(b) simply by the JDOS, thus neglecting plasmonic and other correlation effects, is justified by the large kinetic energy of the Auger electron, ruling out matrix-element effects in the considered energy window. Particularly, dynamical screening effects are strongly suppressed for a swift Auger electron due to the momentum-dependence of the density-density response function.

The accurate description of these central ingredients for describing DPE endorses the predictive power of the current theory. Full details on the calculations is provided by the supplementary information.

## Additional Information

**How to cite this article**: Schüler, M. *et al.* Electron pair escape from fullerene cage via collective modes. *Sci. Rep.* **6**, 24396; doi: 10.1038/srep24396 (2016).

## Supplementary Material

Supplementary Information
