# Corrigendum: Electron pair escape from fullerene cage via collective modes

**DOI:** 10.1038/srep31677

**Published:** 2016-08-25

**Authors:** Michael Schüler, Yaroslav Pavlyukh, Paola Bolognesi, Lorenzo Avaldi, Jamal Berakdar

Scientific Reports 6: Article number: 24396; 10.1038/srep24396published online: 04 18 2016; updated: 08 25 2016

The original version of this Article contained a typographical error in the spelling of the author Paola Bolognesi, which was incorrectly given as Paolo Bolognesi. This has now been corrected in the PDF and HTML versions of the Article.

