# Supplementary material for: Electron pair escape from fullerene cage via collective modes
Source: Sci Rep. 2016 Apr 18;6:24396. doi: 10.1038/srep24396 (PMC4834545; doi:10.1038/srep24396)
Supplement: Supplementary Information [file srep24396-s1.pdf]

Electron pair escape from fullerene cage via collective modes – supplementary information

M. Schüler,* Y. Pavlyukh, and J. Berakdar
Institut für Physik, Martin-Luther-Universität Halle-Wittenberg, 06099 Halle, Germany

P. Bolognesi and L. Avaldi
CNR-ISM, Area della Ricerca di Roma 1, CP10, 00016 Monterotondo Scalo, Italy

I. DFT CALCULATIONS

We performed density-functional theory (DFT) calculations using the Octopus package [1] for obtaining the Kohn-Sham (KS) eigenvalues ϵ_i and bound orbitals $\psi_i(\mathbf{r})$, which serve as the basis set for the subsequent computations. The C_{60} molecule has been represented by its standard icosahedral geometry ($r_{\text{C-C}} = 1.445$ Å, $r_{\text{C=C}} = 1.390$ Å). The core electrons were incorporated by a pseudopotential for the remaining 240 valence electrons. For the exchange-correlation (xc) functional, we have used the local-density approximation (LDA) and added self-interaction corrections (SIC) for restoring the correct asymptotics, a feature that is essential for computing scattering (unbound) states. The simulation was carried out on uniform grid with 0.2 Å spacing confined to a sphere with radius 10 Å. In what follows we use atomic units unless stated otherwise.

The KS orbitals $\psi_i(\mathbf{r})$ were subsequently projected on spherical harmonics $Y_{\ell m}(\hat{\mathbf{r}})$. We found that assigning the dominant contribution with respect to $\ell = \ell_i$ is an excellent approximation [2]. Hence, the expansion of the bound states reads

$$\psi_i(\mathbf{r}) = \sum_{|m| \geq \ell_i} \frac{\phi_{im}(r)}{r} Y_{\ell_i m}(\hat{\mathbf{r}}) \ . \tag{1}$$

In order to compute scattering wave functions orthogonal to eq. (1) we expressed the orbital-averaged KS potential $V^{\text{KS}}(\mathbf{r})$ in terms of a symmetry-adapted function (SAF) representation, that is

$$V^{\text{KS}}(\mathbf{r}) = \sqrt{4\pi} \sum_k V_k^{\text{KS}}(r) I_k(\hat{\mathbf{r}}) \ . \tag{2}$$

The SAFs themselves are eigenfunctions of $\hat{L}^2$ and are linear combinations of the spherical harmonics,

$$I_k(\hat{\mathbf{r}}) = \sum_m C_m^k Y_{\ell_k m}(\hat{\mathbf{r}}) \ , \tag{3}$$

where the symmetry coefficients C_m^k depend on the orientation. The lowest SAFs $k = 1, 2, 3 \ldots$ correspond to angular momentum $\ell_1 = 0$, $\ell_2 = 6$, $\ell_3 = 10, \ldots$; thus the lowest-order correction to a spherically symmetric potential already involves $\ell = 6$. The KS potential in SAF representation is shown in fig. 1.

The SIC scheme allows to restore the correct asymptotic $1/r$ behavior of the KS potential, that usually not given by simple functionals such as LDA alone. For a smooth transition into the asymptotic regime, we replace the numerical values for $V_k^{\text{KS}}(r)$ for $r > r_c = 7.5$ Å by

$$\begin{aligned} V_1^{\text{KS}}(r) &= V_1^{\text{KS}}(r_c) e^{-\eta_1(r-r_c)} - \frac{1 - e^{-\eta_1(r-r_c)}}{r} \ , \\ V_k^{\text{KS}}(r) &= V_k^{\text{KS}}(r_c) e^{-\eta_k(r-r_c)} \ , \ k \geq 2 \end{aligned} \tag{4}$$

and determined the parameters η_k such that the curves are continued smoothly. In fact, the analytical form eq. (4) is very close to the numerical values, but further allows us to extrapolate the KS potential to $r > 10$ Å.

We performed the same procedure for the KS potential of the singly charged C_{60}^+ molecule, imposing $V_1^{\text{KS}}(r) \sim -2/r$.

II. DYNAMICAL SCREENING

In our recent publication [3] we have characterized the retarded density-density response function $\chi^{\text{R}}(\mathbf{r}, \mathbf{r}'; \omega)$ of the C_{60} molecule in terms of a multipole expansion based on *ab initio* calculations. For spherical symmetry, one can expand

$$\chi^{\text{R}}(\mathbf{r}, \mathbf{r}'; \omega) = \sum_{LM} \chi_L^{\text{R}}(r, r'; \omega) Y_{LM}(\hat{\mathbf{r}}) Y_{LM}^*(\hat{\mathbf{r}}') \ . \tag{5}$$

Eq. (5) can be regarded as a very good approximation for systems with small deviation from spherical symmetry. Even

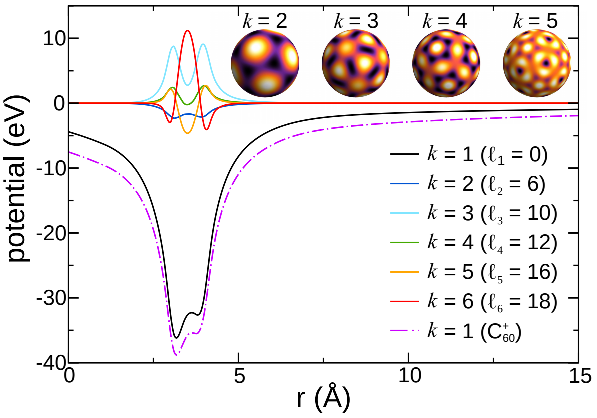

FIG. 1. The orbital-averaged KS potential in SAF representation (full lines). For $r > 7.5$ Å, the extrapolation eq. (4) is used. Insets: graphical representation of SAFs $I_k(\hat{\mathbf{r}})$. Dot-dashed line: spherically averaged ($k = 1$) KS potential of the ionized C_{60}^+ molecule.

* michael.schueler@physik.uni-halle.de

though $\chi^{\mathrm{R}}(\mathbf{r},\mathbf{r}';\omega)$ comprises all many-body excitations, the contribution of plasmons clearly dominates the spectrum at moderate energies ($\omega \sim 10\ldots40$ eV). The plasmon modes in turn can be classified by a radial quantum number ν and angular momentum quantum numbers (L, M). We distinguish between volume (V) modes ($L = 0$), symmetric surface (SS) modes ($\nu = 1, L = 1, 2, \ldots$) and anti-symmetric surface (AS) modes ($\nu = 2, L = 1, 2, \ldots$). The model for the response function is then constructed by restricting the radial dependence $\chi^{\mathrm{R}}_L(r, r';\omega)$ (cf. (5)) to

$$\chi^{\mathrm{R}}_L(r, r';\omega) = \sum_{\nu=1,2} B^{\mathrm{R}}_{\nu L}(\omega) R_{\nu L}(r) R_{\nu L}(r') \ . \tag{6}$$

Here, $R_{\nu L}(r)$ is the radial part of the respective fluctation density (or transition density), and

$$B^{\mathrm{R}}_{\nu L}(\omega) = \frac{2\omega_{\nu,L}}{(\omega + \mathrm{i}\Gamma_{\nu,L})^2 - \omega^2_{\nu L}} \ , \tag{7}$$

where $\omega_{\nu,L}$ now denotes the plasmon frequency and $\Gamma_{\nu,L}$ the peak width. The radial functions $R_{\nu L}(r)$ and the spectral parameters are adopted from ref. 3.

With the density-density response function at our disposal, the dynamical part of the screened interaction can be computed via

$$\delta W^{\mathrm{R}}(\mathbf{r},\mathbf{r}';\omega) = \int d\mathbf{r}_1 \int d\mathbf{r}_2\, v(\mathbf{r}-\mathbf{r}_1)\chi^{\mathrm{R}}(\mathbf{r}_1,\mathbf{r}_2;\omega) v(\mathbf{r}_2 - \mathbf{r}') \ , \tag{8}$$

where $v(\mathbf{r}-\mathbf{r}') = |\mathbf{r}-\mathbf{r}'|^{-1}$ is the bare Coulomb interaction. The time-ordered screened interaction is obtained from equation analogous to eq. (8) using the time-ordered $\chi(\mathbf{r},\mathbf{r}';\omega)$ in place of the retarded response according to the following symmetry relations [4]: $\mathrm{Re}\{\chi(\omega)\} = \mathrm{Re}\{\chi^{\mathrm{R}}(\omega)\}$, $\mathrm{Im}\{\chi(\omega)\} = \mathrm{sign}(\omega)\mathrm{Im}\{\chi^{\mathrm{R}}(\omega)\}$ (spatial arguments are dropped for brevity).

As the response function describes a spherically symmetric system, the screened interaction possesses this property, too, and is thus most conveniently expanded as

$$\delta W(\mathbf{r},\mathbf{r}';\omega) = \sum_{LM} \delta w_L(r, r';\omega) Y_{LM}(\hat{\mathbf{r}}) Y^*_{LM}(\hat{\mathbf{r}}') \ . \tag{9}$$

The integration in eq. (8) can be performed in the spherical basis by defining

$$v_{\nu,L}(r) = \frac{4\pi}{2L+1}\int_0^\infty dr'\, g_L(r, r') R_{\nu L}(r') \tag{10}$$

with $g_L(r, r') = r^L_</r^{L+1}_>$. Hence, we can express the radial part of the screened interaction by

$$\delta w_L(r, r';\omega) = \sum_\nu B_{\nu L}(\omega) v_{\nu L}(r) v_{\nu L}(r') \ . \tag{11}$$

The formation of the final two-particle scattering state involves higher-order effects such as the infinite summation of ladder diagrams in the (T-matrix approximation) [5], as discussed below. In the presence of a medium and bound states in particular, this summation coalesces in additional screening effects [6, 7]. This is accounted for in an approximate fashion by modifying the frequency-independent part of the effective two-electron interaction into a Thomas-Fermi-like form:

$$W^{\mathrm{eff}}(\mathbf{r},\mathbf{r}';\omega) = \frac{e^{-\lambda|\mathbf{r}-\mathbf{r}'|}}{|\mathbf{r}-\mathbf{r}'|} + \delta W(\mathbf{r},\mathbf{r}';\omega) \ . \tag{12}$$

Its functional form is dependent on λ accommodating three effects: a) the static screening which is difficult to obtain from TDDFT because we restrict the sum in (6) to well pronounced collective excitations at higher energies; b) local-field effects relevant for large momentum transfer, describing exchange-correlation hole and expressible in terms of the local field factors [4], and c) post-collision effects describing the interaction of doubly ionized target with the two electrons in the continuum. There are partial solutions addressing each of these cases, however, no general treatment is available. Thus, we retain λ as a parameter, whose numerical value is discussed below.

The Thomas-Fermi potential can be expanded in spherical harmonics

$$\frac{e^{-\lambda|\mathbf{r}-\mathbf{r}'|}}{|\mathbf{r}-\mathbf{r}'|} = 8\lambda \sum_{LM} i_L(\lambda r_<) k_L(\lambda r_>) Y_{LM}(\hat{\mathbf{r}}) Y^*_{LM}(\hat{\mathbf{r}}) \ , \tag{13}$$

in terms of modified spherical Bessel functions [8].

III. DOUBLE PHOTOEMISSION YIELD: DIAGRAM EVALUATION

The central quantity for double photoemission (DPE) is the two-electron current $J_{\mathbf{k}_1,\mathbf{k}_2}$, defined as the number of electrons with momenta $\mathbf{k}_1$ and $\mathbf{k}_2$ detected *in coincidence* per unit of time. This quantity is directly proportional to the actual number of recorded electron pairs in the experiment. For the large number of internal coupling and scattering channels, computing the two-electron photocurrent is a formidable task for interacting system. In our recent publication [9] we developed a systematic approach for calculating $J_{\mathbf{k}_1,\mathbf{k}_2}$ by an expansion in Feynman diagrams. This technique has also the advantage that we sort the contributions to the photocurrent according to differential physical mechanisms, such as extrinsic plasmon losses or screening of the radiation field. Here we explicitly evaluate the lowest-order (in the screened interaction) *knock-out-type* diagram for DPE, shown in fig. 2(a). An alternative, yet equivalent approach is given by a diagrammatic expansion of the two-body transition dipole matrix element and subsequent application of Fermi's Golden rule [10]. Note that we concentrate on *final-state* correlations here, i. e., initial-state correlation effects such as shake-off processes are not taken into account. This is justified by the fact that carbon-based materials are weakly correlated in terms of ground-state properties, while the strong plasmonic resonances mostly affect photoelectrons.

The constituents of the diagram fig. 2(a) are the fully-interacting Green's function (GF) G and the screened interaction W, comprising the bare Coulomb interaction and dynamical screening effects, and the operator of light-matter interaction (in dipole approximation) $\hat{d}$. The dots on the right-hand

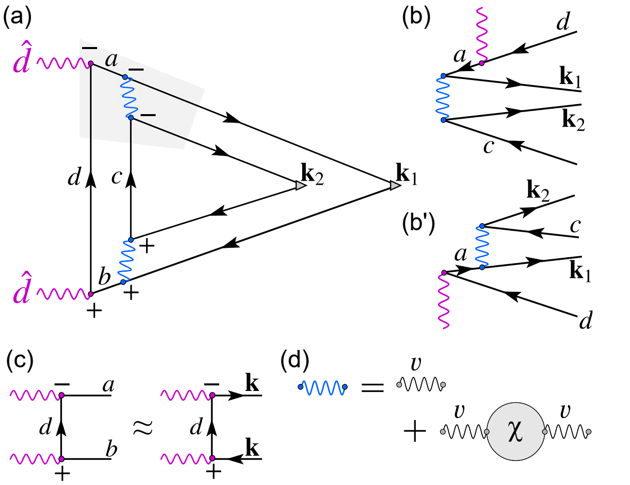

FIG. 2. (a) The lowest-order knock-out diagram for the DPE mediated by the screened interaction W (indicated by the blue wavy line). We assume a weak incident field (light-matter-interaction operator $\hat{d}$) with photon energy ω (violet lines). Gray triangles denote the projection onto the detector states. (b,b′) Corresponding diagrams for the two-body dipole matrix element (Goldstone diagrams [11]) according to ref. 10, which are equivalent [12] to the shaded area in the diagram (a). (c) Diagonal approximation for the optical absorption part leading to further simplifications (for justification see text). (d) Diagrammatic representation of the screened interaction W in terms of the bare Coulomb interaction v and the full density-density response function χ.

side labeled by $\mathbf{k}_1$ and $\mathbf{k}_2$, respectively, represent the detected electrons (such that their momentum is fixed). Here, $\mathbf{k}_1$ is the primary electron, while $\mathbf{k}_2$ stands for the secondary electron that is emitted after the internal scattering process mediated by W has taken place. After the evaluation of the diagram on fig. 2(a) the symmetrization accounting for the indistinguishability of the two electrons is performed.

The full GF needs to be calculated in some applicable approximation. In particular for the time-ordered GF we use the mean-field expression:

$$\hat{G}(\epsilon) = \sum_{a\in\mathrm{occ}} \frac{|\psi_a\rangle\langle\psi_a|}{\epsilon - \epsilon_a - \mathrm{i}\delta} + \sum_{a\in\mathrm{virt}} \frac{|\psi_a\rangle\langle\psi_a|}{\epsilon - \epsilon_a + \mathrm{i}\delta} \, , \; \delta \to 0^+ \, , \tag{14}$$

where $|\phi_a\rangle$ are the mean-field eigenstates with eigenvalues ϵ_a. Other propagators (anti-time-ordered, greater etc.) are defined consistently. All states with $\epsilon_a < \mu$ are occupied, whereas states with $\epsilon_a \geq \mu$ are empty (virtual). All ingredients to eq. (14) have been determined by the DFT calculation (see sec. I). Using eq. (14) the DPE photocurrent for the diagram fig. 2(a) reads

$$\begin{aligned} J_{\mathbf{k}_1\mathbf{k}_2} \propto & \int_{-\infty}^{\mu} \mathrm{d}\zeta \int_{-\infty}^{\mu} \mathrm{d}\bar{\zeta} \int_0^\infty \mathrm{d}\xi \, \delta(\xi + \epsilon_{\mathbf{k}_1} - \omega - \zeta) \\ & \times \sum_{abcd} \frac{1}{\omega + \zeta - \epsilon_a - \mathrm{i}\delta} \frac{1}{\omega + \zeta - \epsilon_b + \mathrm{i}\delta} \\ & \times \langle\psi_a|\hat{d}|\psi_d\rangle\langle\psi_b|\hat{d}|\psi_d\rangle^* W^*_{\mathbf{k}_2\mathbf{k}_1cb}(\xi) W_{\mathbf{k}_2\mathbf{k}_1ca}(\xi) \\ & \times \delta(\bar{\zeta} - \epsilon_c)\delta(\epsilon_{\mathbf{k}_2} - \xi - \bar{\zeta})\delta(\zeta - \epsilon_d) \, . \end{aligned} \tag{15}$$

Here we have defined the two-particle matrix elements of the screened interaction according to

$$W_{abcd}(\xi) = \int \mathrm{d}\mathbf{r} \int \mathrm{d}\mathbf{r}' \psi^*_a(\mathbf{r})\psi^*_b(\mathbf{r}')W(\mathbf{r},\mathbf{r}';\xi)\psi_d(\mathbf{r}')\psi_c(\mathbf{r}) \, . \tag{16}$$

The labelling by the indices is identical to fig. 2. The main contribution to the photocurrent arises when the denominators in the second line in eq. (15) approach zero. In the limit $\delta \to 0$, the current is sharply peaked at $\epsilon_a = \epsilon_b = \omega + \zeta$. Assuming a small degree of degeneracy for the intermediate states a, b, it is reasonable to approximate eq. (15) by just including the $a = b$ in the sum. Further justification is given below. This assumption simplifies the expression significantly. Let us define the distribution function

$$f_a(\zeta) = \sum_d \left|\langle\psi_a|\hat{d}|\psi_d\rangle\right|^2 \delta(\zeta - \epsilon_d) \, . \tag{17}$$

For a non-interacting system, eq. (17) is nothing else (up to a prefactor) but the single-ionization cross section (for continuum states a). In any case we can interpret $f_a(\zeta)$ as the distribution of photo-excited electrons right after absorbing one photon. Inserting eq. (15) and assuming $a = b$ results in

$$\begin{aligned} J_{\mathbf{k}_1\mathbf{k}_2} \propto & \int_{-\infty}^{\mu} \mathrm{d}\zeta \int_{-\infty}^{\mu} \mathrm{d}\bar{\zeta} \int_0^\infty \mathrm{d}\xi \, \delta(\xi + \epsilon_{\mathbf{k}_1} - \omega - \zeta) \\ & \times \sum_{ac} \frac{1}{(\omega + \zeta - \epsilon_a)^2 + \delta^2} \left|W_{\mathbf{k}_2\mathbf{k}_1ca}(\xi)\right|^2 f_a(\zeta) \\ & \times \delta(\bar{\zeta} - \epsilon_c)\delta(\epsilon_{\mathbf{k}_2} - \xi - \bar{\zeta}) \, . \end{aligned} \tag{18}$$

According to eq. (18), the mechanism of the plasmon-assisted DPE can be understood in two steps: (i) excitation of states a and (ii) interaction of two electrons (ac) via the screened interaction resulting in two electrons in the continuum ($\mathbf{k}_1\mathbf{k}_2$) (fig. 2(b)). For sufficiently large photon energy ω, the photo-excited states labeled by a are situated above the ionization threshold (the states c are bound). Hence we relabel $a \to \mathbf{k}$, $c \to n$. Performing the integration over $\bar{\zeta}$, the simplified current attains the form

$$\begin{aligned} J_{\mathbf{k}_1\mathbf{k}_2} \propto & \int_{-\infty}^{\mu} \mathrm{d}\zeta \int_0^\infty \mathrm{d}\xi \, \delta(\xi + \epsilon_{\mathbf{k}_1} - \omega - \zeta) \\ & \times \sum_{n\in\mathrm{occ}} \delta(\epsilon_{\mathbf{k}_2} - \xi - \epsilon_n) \int \mathrm{d}^3\mathbf{k} \frac{1}{(\omega + \zeta - \epsilon_{\mathbf{k}})^2 + \delta^2} \\ & \times \left|W_{\mathbf{k}_2\mathbf{k}_1n\mathbf{k}}(\xi)\right|^2 f_{\mathbf{k}}(\zeta) \, . \end{aligned} \tag{19}$$

As the intermediate states haven been identified with photoelectron and thus particle states, the process described by the diagram fig. 2 (b) can be ommitted, leaving fig. 2 (b′) as the dominant contribution.

In order to factor out the distribution function (17), we assumed that the intermediate-state quantum numbers are identical: $\mathbf{k} \approx \mathbf{k}'$. Actually, only $|\mathbf{k}| = |\mathbf{k}'|$ can be justified rigorously. In other words, interference effects in the angular channel are excluded by the simplification (represented by the diagram fig. 2(c)) leading to eq. (17). While this approximation

will certainly not suffice to correctly predict the angular distribution of the photoelectrons in a DPE experiment, the loss of angular coherence only weakly affects the angle-integrated DPE yield, the quantity which we will focus on.

A. Application to C_{60} molecule

Now we focus on the situation as in the experiment where the angle of the photoelectron is not detected. The angle-integrated photocurrent

$$J(\epsilon_{\mathbf{k}_1}, \epsilon_{\mathbf{k}_2}) = \int d\hat{\mathbf{k}}_1 \int d\hat{\mathbf{k}}_2 J_{\mathbf{k}_1\mathbf{k}_2} \quad (20)$$

can be computed starting from eq. (19). We have to integrate over the angles $\hat{\mathbf{k}}_1$ and $\hat{\mathbf{k}}_2$, the momenta of the intermediate state ($\mathbf{k}$) as well as the six-dimensional two-body matrix element with respect to the screened interaction. Furthermore, the average over all possible orientations of the molecule needs to be performed to reflect the gas-phase experiment. This is a very demanding task entailing a number of approximations to obtain manageable expressions.

First we exploit the almost perfect spherical symmetry to split the average over the orientations of the complete expression into the product of orientation-averaged screened interaction and photoemission probability:

$$\boxed{\left\langle \left|W_{\mathbf{k}_2\mathbf{k}_1 n\mathbf{k}}(\xi)\right|^2 f_{\mathbf{k}}(\zeta)\right\rangle_c \approx \left\langle \left|W_{\mathbf{k}_2\mathbf{k}_1 n\mathbf{k}}(\xi)\right|^2\right\rangle_c \langle f_{\mathbf{k}}(\zeta)\rangle_c \, .}$$

The orientation-averaged distribution can be set in relation to the differential cross section $d\sigma/d\hat{\mathbf{k}}$ for single photoemission (SPE) [5]:

$$\frac{d\sigma}{d\hat{\mathbf{k}}} = 4\pi\alpha_0^2 \omega k \langle f_{\mathbf{k}}(\epsilon_{\mathbf{k}} - \omega)\rangle_c \, , \quad (21)$$

where α_0 denote the fine-structure constant. Orientation-averaged cross section is given by the Cooper-Zare [13] form:

$$\frac{d\sigma}{d\hat{\mathbf{k}}} = \frac{\sigma_0(\epsilon_{\mathbf{k}}, \omega)}{4\pi}\left[1 + \beta(\epsilon_{\mathbf{k}})P_2(\cos\theta)\right] \, . \quad (22)$$

Here, $\sigma_0(\epsilon_{\mathbf{k}}, \omega)$ stands for the partial cross section for the emission of one the photoelectron with energy $\epsilon_{\mathbf{k}}$ upon absorption of one photon ω, P_2 the second Legendre polynomial and $\beta(\epsilon_{\mathbf{k}}, \omega)$ the angular asymmetry or β–parameter. The angle θ is measured between $\mathbf{k}$ and the polarization of the incident radiation. The total cross section σ_0 and the asymmetry parameter β are known from various experimental and theoretical works, such that we can compare for reference. The parametrization eq. (22) dictates the analogous dependence,

$$\langle f_{\mathbf{k}}(\zeta)\rangle_c = f^{(0)}(k,\zeta)Y_{00}(\hat{\mathbf{k}}) + f^{(2)}(k,\zeta)Y_{20}(\hat{\mathbf{k}}) \, ,$$

which allows us to simplify

$$\int d\hat{\mathbf{k}}_1 \int d\hat{\mathbf{k}}_2 \int d\hat{\mathbf{k}} \, \langle |W_{\mathbf{k}_2\mathbf{k}_1 n\mathbf{k}}(\xi)|^2\rangle_c \langle f_{\mathbf{k}}(\zeta)\rangle_c \equiv \mathcal{U}^{(0)}_{k_2k_1nk}(\xi) f^{(0)}(k,\zeta) + \mathcal{U}^{(2)}_{k_2k_1nk}(\xi) f^{(2)}(k,\zeta) \, ,$$

with

$$\mathcal{U}^{(0)}_{k_2k_1nk}(\xi) = \frac{1}{\sqrt{4\pi}} \int d\hat{\mathbf{k}}_1 \int d\hat{\mathbf{k}}_2 \int d\hat{\mathbf{k}} \, \langle |W_{\mathbf{k}_2\mathbf{k}_1 n\mathbf{k}}(\xi)|^2\rangle_c \quad (23)$$

and

$$\mathcal{U}^{(2)}_{k_2k_1nk}(\xi) = \int d\hat{\mathbf{k}}_1 \int d\hat{\mathbf{k}}_2 \int d\hat{\mathbf{k}} \, \langle |W_{\mathbf{k}_2\mathbf{k}_1 n\mathbf{k}}(\xi)|^2\rangle_c Y_{20}(\hat{\mathbf{k}}) \, . \quad (24)$$

It is straightforward to show that $\mathcal{U}^{(2)}_{k_2k_1nk}(\xi)$ vanishes when summing over closed electronic shells (it follows from properties of the Gaunt coefficients). For the C_{60} molecule, the highest occupied molecular orbital (HOMO) contains 10 electrons, which is much smaller than the total number of 240 valence electrons. For this reason, the contribution to the photocurrent related to the asymmetry parameter are negligible.

In the next steps we describe how to calculate the angle-integrated two-body matrix element eq. (23) with the screened interaction expanded according to eqs. (9),(11) and bound orbitals as in eq. (1). A major simplification for the subsequent angular integration can be achieved if the continuum orbitals are expanded into

$$\psi_{\mathbf{k}}(\mathbf{r}) = \sum_{\ell m} z_\ell \frac{\phi_{k\ell}(r)}{r} Y_{\ell m}(\hat{\mathbf{r}}) Y^*_{\ell m}(\hat{\mathbf{k}}) \, . \quad (25)$$

Here, $z_\ell = \mathrm{i}^\ell e^{-\mathrm{i}\eta_\ell}$ with $\eta_\ell = \eta_\ell(k)$ denoting the Coulomb phase shift. Note that eq. (25) applies to spherically symmetric systems. Thus, $\psi_{\mathbf{k}}(\mathbf{r})$ is computed with respect to the spherically-averaged KS potential $V^{\mathrm{KS}}_{k=1}(r)$ (cf. eq. (2)).

Let us further abbreviate the Gaunt coefficients by

$$G(\ell_1 m_1 \ell_2 m_2|LM) \equiv \int d\hat{\mathbf{r}} \, Y^*_{LM}(\hat{\mathbf{r}}) Y_{\ell_1 m_1}(\hat{\mathbf{r}}) Y_{\ell_2 m_2}(\hat{\mathbf{r}}) \, , \quad (26)$$

and express the Clebsch-Gordan coefficients in terms of the Wigner 3j-symbol. The Gaunt coefficients are then given by

$$G(\ell_1 m_1 \ell_2 m_2|LM) = (-1)^M \sqrt{\frac{c_{\ell_1}c_{\ell_2}c_L}{4\pi}} \begin{pmatrix} \ell_1 & \ell_2 & L \\ 0 & 0 & 0 \end{pmatrix} \begin{pmatrix} \ell_1 & \ell_2 & L \\ m_1 & m_2 & -M \end{pmatrix},$$

where $c_\ell = 2\ell + 1$. The matrix element of the screened interaction can thus be written as

$$W_{\mathbf{k}_2\mathbf{k}_1 n\mathbf{k}}(\xi) = \sum_{\ell_1 m_1} \sum_{\ell_2 m_2} \sum_{\ell' m'} \sum_{|m_n|\geq \ell_n} \sum_{LM} z^*_{\ell_2} z^*_{\ell_1} z_{\ell'} G(\ell_n m_n LM|\ell_2 m_2) \times G(\ell_1 m_1 LM|\ell' m')(\phi_{k_2\ell_2}\phi_{k_1\ell_1}|w_L(\xi)|\phi_{k\ell'}\phi_{nm_n}) \times Y_{\ell_1 m_1}(\hat{\mathbf{k}}_1) Y_{\ell_2 m_2}(\hat{\mathbf{k}}_1) Y^*_{\ell' m'}(\hat{\mathbf{k}}') \, ,$$

where the reduced two-body matrix element reads

$$(\phi_{k_2\ell_2}\phi_{k_1\ell_1}|w_L(\xi)|\phi_{k\ell'}\phi_{nm_n}) = \int_0^\infty dr \int_0^\infty dr' \, \phi^*_{k_2\ell_2}(r)\phi^*_{k_1\ell_1}(r') \times w_L(r,r';\xi)\phi_{k\ell'}(r')\phi_{nm_n}(r) \, . \quad (27)$$

Using the algebraic properties of the Clebsch-Gordan coefficients [14, 15],

$$\sum_{m_1 m'} G(\ell_1 m_1 LM|\ell' m')G(\ell_1 m_1 L' M'|\ell' m') = \frac{c_{\ell_1} c_{\ell'}}{4\pi} \begin{pmatrix} \ell_1 & L & \ell' \\ 0 & 0 & 0 \end{pmatrix}^2 \times \delta_{LL'} \delta_{MM'}$$

and

$$\sum_{Mm_2} G(\ell_n m_n LM|\ell_2 m_2)^2 = \frac{c_L c_{\ell_2}}{4\pi} \begin{pmatrix} L & \ell_n & \ell_2 \\ 0 & 0 & 0 \end{pmatrix}^2$$

we obtain

$$\mathcal{U}^{(0)}_{k_2 k_1 nk}(\xi) = \frac{1}{(4\pi)^{5/2}} \sum_{\ell_1,\ell_2,\ell'} \sum_{|m_n|\geq \ell_n} \sum_{L} c_{\ell_1} c_{\ell_2} c_{\ell'} c_L \times \begin{pmatrix} \ell_1 & L & \ell' \\ 0 & 0 & 0 \end{pmatrix}^2 \begin{pmatrix} L & \ell_n & \ell_2 \\ 0 & 0 & 0 \end{pmatrix}^2 \times \left| \left(\phi_{k_2 \ell_2} \phi_{k_1 \ell_1} \middle| w_L(\xi) \middle| \phi_{k\ell'} \phi_{n m_n} \right) \right|^2 . \quad (28)$$

In summary, the assumptions and simplifications employed in this subsection allow for casting the angle-integrated two-electron photocurrent eq. (20) into the simple form

$$\boxed{J(\epsilon_{\mathbf{k}_1}, \epsilon_{\mathbf{k}_2}) \propto \frac{1}{\omega} \int_{-\infty}^{\mu} d\zeta \int_0^{\infty} d\xi\, \delta(\xi + \epsilon_{\mathbf{k}_1} - \omega - \zeta) \times \sum_{n\in \mathrm{occ}} \delta(\epsilon_{\mathbf{k}_2} - \xi - \epsilon_n) \int_0^{\infty} dk\, k\, \sigma_0(\epsilon_{\mathbf{k}}, \omega) \times \mathcal{U}^{(0)}_{k_2 k_1 nk}(\xi) \delta(\omega + \zeta - \epsilon_{\mathbf{k}}) ,} \quad (29)$$

where we have taken the limit $\delta \to 0$ in eq. (19) and dropped all unnecessary prefactors.

B. Density of states and single ionization cross section

The single photoemission cross section is an important ingredient of the two-electron photocurrent eq. (29). We calculated the cross section using the driven-scattering technique described in detail in ref. 16. It allows for efficient computing of the scattering amplitude and thus of the partial cross section for any multi-channel one-dimensional Schrödinger equation without calculating the scattering wave functions explicitly. Our result for the total cross section

$$\sigma_{\mathrm{tot}}(\omega) = \int_0^{\infty} d\epsilon\, \sigma_0(\epsilon, \omega) \quad (30)$$

is presented in fig. 3(a) and compared (on absolute scale) to the theoretical work of Colvita [17] and the experiment of Reinköster [18].

The critical quantity to achieve convergence is the number of SAFs included to expand $V^{\mathrm{KS}}(\mathbf{r})$. We remark that the accuracy of our SPE cross section shows some deviation in the region below 30 eV, whereas we reached converged results for higher photon energies. However, since the threshold for the overall DPE process we are considering is around 40 eV, taking 4 SAFs (corresponds to $\ell_{\max} = 12$) into account (fig. 3(a)) already yields quite precise results for the energy region of interest.

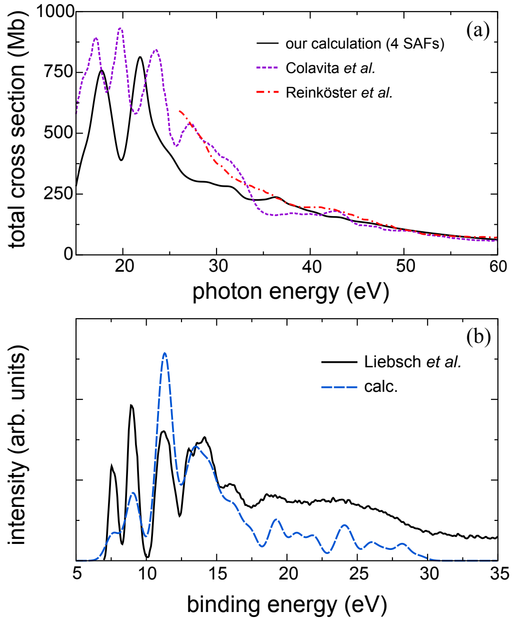

FIG. 3. (a) The total SPE cross section as a function of the photon energy for 4 SAFs included to represent the KS potential (thick lines). Dot-dashed line: experimental data from ref. 18, dotted line: theory result from ref. 17. (b) The SPE binding energy spectrum $S(\epsilon)$ at $\omega = \hbar 65$ eV photon energy, calculated with the driven-scattering technique (blue dashed line) along with experimental results from ref. 19.

In order to correct the deficiencies of DFT due to the approximations to the xc functional when describing SPE spectra, we rescaled the KS eigenvalues to match experimental data. In particular, we compute the bindining energy spectrum

$$S(\epsilon) \propto \langle f_{\mathbf{k}}(-\epsilon)\rangle_c . \quad (31)$$

at fixed photon energy $\hbar\omega = 65$ eV and compare it to the experimental results from Liebsch *et al.* [19]. The comparison of our calculated spectrum and the experiment (fig. 3(b)) demonstrates that all spectral features coincide. The HOMO position is adjusted to coincide with the (negative) experimental ionization potential (IP) from ref. 20 (IP(C_{60}) = 7.59 eV). Note that the experiment has some background signal, as the intensity does not drop to zero at high binding energy (there are not states to ionize), indicating secondary processes.

C. Correlation and charge correction for singly ionized molecule

Inspecting again eq. (29) we realize that $\delta(\epsilon - \epsilon_n)$ (first term in second line corresponding to the inner vertical line in fig. 2(a)) is nothing else but the spectral function of the singly ionized system. However the KS eigenvalues ϵ_n of the

neutral C_{60} molecule ignore orbital relaxation effects, which are of importance for a correct energy dependence including the double ionization threshold. This effect is accounted for by introducing the shift $\Delta = \mathrm{IP}(C_{60}^{+}) - \mathrm{IP}(C_{60})$. The respective IPs are taken from experiments [20, 21], yielding $\Delta = 3.81$ eV. Replacing the Dirac delta containing the bound states in eq. (29) by $\widetilde{A}_n(\epsilon) = \delta(\epsilon - \epsilon_n - \Delta)$ thus accounts for these correlation effects.

D. Test calculations & screening parameter

In order to investigate the importance of the individual ingredients entering the total DPE yield eq. (29) we tested a number of simplifications. Ignoring the frequency dependence of the two-body matrix elements, $\mathcal{U}^{(0)}_{k_2 k_1 n k}(\xi) \approx \mathcal{U}^{(0)}_{k_2 k_1 n k}$ allows for carrying out the ξ-integration, yielding

$$J(\epsilon_{\mathbf{k}_1}, \epsilon_{\mathbf{k}_2}) \propto \frac{1}{\omega} \sum_{n\in \mathrm{occ}} \int_0^\infty \mathrm{d}k\, k\, \mathcal{U}^{(0)}_{k_2 k_1 n k}\, \sigma_0(\epsilon_{\mathbf{k}}, \omega) \times \widetilde{A}_n(\epsilon_{\mathbf{k}_1} + \epsilon_{\mathbf{k}_2} - \epsilon_{\mathbf{k}}) \,. \qquad (32)$$

The next level of approximation is achieved by ignoring matrix-element effects of the two-body interaction, reducing eq. (32) to

$$J(\epsilon_{\mathbf{k}_1}, \epsilon_{\mathbf{k}_2}) \propto \frac{1}{\omega} \sum_{n\in \mathrm{occ}} \int_0^\infty \mathrm{d}k\, k\, \sigma_0(\epsilon_{\mathbf{k}}, \omega) \widetilde{A}_n(\epsilon_{\mathbf{k}_1} + \epsilon_{\mathbf{k}_2} - \epsilon_{\mathbf{k}}) \,. \qquad (33)$$

Finally, neglecting optical matrix element effects simplifies the SPE cross section to

$$\sigma_0(\epsilon_{\mathbf{k}}, \omega) \propto \omega \sum_{m\in \mathrm{occ}} \left|\langle \psi_{\mathbf{k}} | \hat{d} | \psi_m \rangle\right|^2 \delta(\epsilon_{\mathbf{k}} - \omega - \epsilon_m) \approx \omega \sum_{m\in \mathrm{occ}} A_m(\epsilon_{\mathbf{k}} - \omega) \,,$$

where $A_m(\epsilon)$ denotes the spectral function of the neutral molecule. The resulting coincidence yield thus amounts to the convolution

$$J(\epsilon_{\mathbf{k}_1}, \epsilon_{\mathbf{k}_2}) \propto \int_0^\infty \mathrm{d}k\, k\, D(\epsilon_{\mathbf{k}} - \omega) \widetilde{D}(\epsilon_{\mathbf{k}_1} + \epsilon_{\mathbf{k}_2} - \epsilon_{\mathbf{k}}) \qquad (34)$$

$$= \int_0^\infty \mathrm{d}\epsilon_{\mathbf{k}}\, D(\epsilon_{\mathbf{k}} - \omega) \widetilde{D}(\epsilon_{\mathbf{k}_1} + \epsilon_{\mathbf{k}_2} - \epsilon_{\mathbf{k}}) \,, \qquad (35)$$

where $D(\epsilon) = \sum_{m\in \mathrm{occ}} A_m(\epsilon)$ (analogously for $\widetilde{D}(\epsilon)$) is the density of occupied states. Eq. (34) is commonly referred to as joint density of states (JDOS) and is the standard quantity to interpret Auger spectra (see main text). The simplifications eq. (32)–(34) are tested against the full expression eq. (29) in fig. 4(a).

As demonstrated by fig. 4(a), the distinct feature of the observed DPE yield (the significant narrowing of the spectrum, which is in strong constrast to the Auger spectrum), is best described by the full expression eq. (29). Hence, all effects, that is the DOS, SPE matrix elements, two-body matrix elements and plasmonic properties, are important for a correct description of the present experimental data. Moreover, the value of the screening parameter λ can be estimated from fig. 4. Tests have shown that for small λ, the effective interaction eq. (12) is dominated by the almost Coulomb-like static part. The dynamical contribution $\delta W(\mathbf{r}, \mathbf{r}'; \xi)$ is negligible in this case, the frequency dependence of the effective interaction plays only a subsidiary role (static regime), and eq. (32) is a very good approximation. The strength of the plasmonic excitations in the C_{60} (as they almost completely exhaust the f-sum rule [22]) however indicates that the effective interaction mediated by the charge-density fluctuations is dominant. This is underpinned by the narrowing of the DPE spectrum as λ is increased. We fix $\lambda = 2.0$ a_B, where the dynamical part $\delta W(\mathbf{r}, \mathbf{r}'; \xi)$ overweighs the static contribution (plasmon regime).

As explained in the text, the tighter DPE spectra are mostly due to the limited energy the plasmonic excitations can deliver to the system in order ionize the second electron. This physical picture is confirmed by fig. 4(b), where a considerable suppression of the emission from the deeper σ states is observed in the plasmon regime. On the contrary, there is no such energy confinement in the static regime.

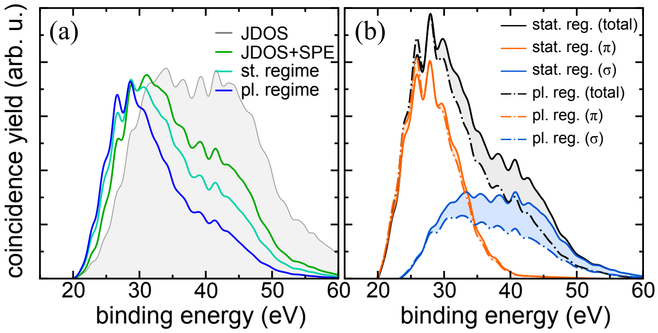

FIG. 4. (a) Test calculations for the DPE yield including different effects: JDOS only (eq. (34)), including SPE matrix elements (eq. (33)), including two-body matrix elements (static regime, eq. (32)), and the full formula (plasmon regime, eq. (29)). (b) Coincidence yield in the static regime (full lines) and in the plasmon regime (dot-dashed lines), resolved in emission of the second electron from σ and π states, respectively. The shaded areas indicate the difference between the two scenarios. For smooth curves, the Dirac-deltas in the spectral functions functions have been replaced by Gaussians with width $\eta = 0.5$ eV.

[1] M. A. L. Marques, A. Castro, G. F. Bertsch, and A. Rubio, Comp. Phys. Commun. **151**, 60 (2003).

[2] Y. Pavlyukh and J. Berakdar, Chem. Phys. Lett. **468**, 313

(2009).
[3] M. Schüler, J. Berakdar, and Y. Pavlyukh, Phys. Rev. A **92**, 021403 (2015).
[4] G. Giuliani and G. Vignale, *Quantum Theory of the Electron Liquid* (Cambridge University Press, 2005).
[5] J. Berakdar, *Concepts of Highly Excited Electronic Systems* (John Wiley & Sons, 2006).
[6] D. O. Gericke, S. Kosse, M. Schlanges, and M. Bonitz, Phys. Rev. B **59**, 10639 (1999).
[7] I. A. Nechaev and E. V. Chulkov, Phys. Rev. B **71**, 115104 (2005).
[8] F. W. J. Olver, D. W. Lozier, R. F. Boisvert, and C. W. Clark, *NIST Handbook of Mathematical Functions* (Cambridge University Press, 2010).
[9] Y. Pavlyukh, M. Schüler, and J. Berakdar, Phys. Rev. B **91**, 155116 (2015).
[10] M. Y. Amusia, *Atomic Photoeffect* (Springer Science & Business Media, 2013).
[11] E. S. Chang and M. R. C. McDowell, Phys. Rev. **176**, 126 (1968).
[12] Y. Pavlyukh, arXiv:1601.04278 [cond-mat] (2016).
[13] J. Cooper and R. N. Zare, J. Chem. Phys. **48**, 942 (1968).
[14] A. R. Edmonds, *Angular Momentum in Quantum Mechanics* (Princeton University Press, 1996).
[15] D. A. Varshalovich and A. N. Moskalev, *Quantum Theory of Angular Momentum: Irreducible Tensors, Spherical Harmonics, Vector Coupling Coefficients, 3nj Symbols* (World Scientific Pub., 1988).
[16] M. Schüler, Y. Pavlyukh, and J. Berakdar, Phys. Rev. A **89**, 063421 (2014).
[17] P. Colavita, G. De Alti, G. Fronzoni, M. Stener, and P. Decleva, Phys. Chem. Chem. Phys. **3**, 4481 (2001).
[18] A. Reinköster, S. Korica, G. Prümper, J. Viefhaus, K. Godehusen, O. Schwarzkopf, M. Mast, and U. Becker, J. Phys. B **37**, 2135 (2004).
[19] T. Liebsch, O. Plotzke, F. Heiser, U. Hergenhahn, O. Hemmers, R. Wehlitz, J. Viefhaus, B. Langer, S. B. Whitfield, and U. Becker, Phys. Rev. A **52**, 457 (1995).
[20] H. Steger, J. Holzapfel, A. Hielscher, W. Kamke, and I. V. Hertel, Chem. Phys. Lett. **234**, 455 (1995).
[21] H. Steger, J. de Vries, B. Kamke, W. Kamke, and T. Drewello, Chem. Phys. Lett. **194**, 452 (1992).
[22] F. Alasia, R. A. Broglia, H. E. Roman, L. Serra, G. Colo, and J. M. Pacheco, J. Phys. B **27**, L643 (1994).
